# Trends in Incidence and Survival of Patients With Primary Effusion Lymphoma in the United States: A Population Based Cohort Study

**DOI:** 10.1002/hon.70168

**Published:** 2026-01-09

**Authors:** John L. Vaughn, Gardenia Taza, Malak Munir, Sravani Rimmalapudi, Narendranath Epperla

**Affiliations:** ^1^ Division of Hematology & Oncology NYU Grossman Long Island School of Medicine Mineola New York USA; ^2^ Karsh Division of Gastroenterology and Hepatology Cedars‐Sinai Medical Center Los Angeles California USA; ^3^ Division of Hematology and Hematologic Malignancies Huntsman Cancer Institute University of Utah Salt Lake City Utah USA

**Keywords:** incidence, primary effusion lymphoma, survival, time period

## Abstract

Primary effusion lymphoma (PEL) is a rare and aggressive B‐cell non‐Hodgkin lymphoma (NHL) that predominantly affects patients with human immunodeficiency virus infection and is strongly associated with human herpes virus 8 (HHV‐8) infection. Due to its rarity, the current understanding of PEL's epidemiology and management is largely derived from case reports and small retrospective studies. Using the SEER‐17 database, we conducted a retrospective analysis of adults with pathologically confirmed primary effusion lymphoma diagnosed between 2001–2021. Patients were stratified into two time periods (2001–2010 and 2011–2021) to assess temporal trends. Age‐adjusted incidence rates, relative survival (RS), overall survival (OS), and lymphoma‐specific survival (LSS) were calculated using flexible parametric survival models. Competing risk analysis was performed to evaluate cumulative incidence of lymphoma‐specific death. Among 236 patients (median age 51 years, 88% male), 82 were diagnosed in 2001–2010 and 154 in 2011–2021. Age‐adjusted incidence rates increased from 1.0 to 1.6 cases per 10,000,000 person‐years between periods (*p* = 0.004). Five‐year RS improved from 21% to 37%, with median OS increasing from 4 to 12 months. On multivariable analysis, the more recent period showed significant improvements in OS (HR = 0.65; 95% CI, 0.44–0.97) and LSS (HR = 0.56; 95% CI, 0.36–0.86), with reduced cumulative incidence of lymphoma‐specific death (HR = 0.49; 95% CI, 0.33–0.74). In our population‐level analysis of PEL, we report a significant improvement in survival outcomes between 2001–2021, likely reflecting advances in both lymphoma treatment and HIV management. However, despite these improvements, OS remains low underscoring the need for prioritizing these patients to clinical trials with novel therapies.

## 1 Introduction

Primary effusion lymphoma (PEL) is a rare type of B‐cell non‐Hodgkin lymphoma (NHL) that was first classified by the World Health Organization (WHO) in 2001 [1]. PEL is characterized by malignant effusions in body cavities usually in the absence of extracavitary masses. Symptoms are dependent on the affected cavities and often include B‐symptoms.

PEL is commonly associated with human immunodeficiency virus (HIV) infection. However, it can also occur in non‐HIV infected individuals including elderly and immunocompromised patients. The detection of Human Herpesvirus‐8 (HHV‐8), also known as Kaposi Sarcoma Herpes Virus, is essential for the diagnosis of PEL [2]. PEL is more commonly reported in Caucasian males (median age of 41 years) [3] and carries a poor prognosis, with a reported 5‐year overall survival of 17% [3]. Although strongly associated with the onset of acquired immunodeficiency syndrome (AIDS), [4]. some studies suggest a positive correlation between HIV status and response to chemotherapy [5]. Despite significant advances in the treatment of aggressive lymphomas over the past decade, it remains unclear whether these improvements have translated to better outcomes for PEL at the population level. Hence, we sought to evaluate the incidence and survival trends in patients with PEL using the Surveillance, Epidemiology, and End Results Program (SEER)‐17 database.

## 2 Methods

### 2.1 Patient Population and Study Design

Patients were included in the study if they were aged 18 years and older with pathologically confirmed primary effusion lymphoma diagnosed in the USA between 2001–2021. The study population was divided into two time periods, time period‐1 (2001–2010) and time period‐2 (2011–2021). These periods were chosen based on a literature review to reflect the wider use of the dose‐adjusted (DA)‐EPOCH regimen (etoposide, prednisone, vincristine, cyclophosphamide and doxorubicin) after 2010. Patients who were diagnosed by autopsy or death were excluded.

The data source was the SEER‐17 database that represents 27% of the US population. The study was conducted in compliance with the Declaration of Helsinki. Since this was a retrospective study using publicly available data, the study was exempt from Institutional Board Review and informed consent was waived.

### 2.2 Outcomes and Covariates

The study outcomes were relative survival (RS), overall survival (OS), lymphoma‐specific survival (LSS), and the cumulative incidence of death from lymphoma (CIF). RS was defined as the ratio of all‐cause survival to expected survival in a comparable group of individuals from the general population. Expected survival was estimated by matching patients in our study to individuals in the general population by age, sex, year, and race using data provided by SEER. OS was defined as the probability of death from any cause following diagnosis of lymphoma. LSS was defined as the probability of survival when lymphoma was considered the only possible cause of death. The CIF was defined as the probability of death from lymphoma in the presence of the competing risk of death from other causes. The study covariates were age, sex, race (White, Black, and Other), stage, documentation of B symptoms, and documentation of whether a patient received chemotherapy for their lymphoma.

### 2.3 Statistical Analysis

Patient characteristics were described using descriptive statistics. Differences between characteristics were tested using the Wilcoxon rank‐sum and chi‐squared tests. Age‐adjusted incidence rates and incidence rate ratios were calculated using SEER*Stat Software version 8.4.4. Counts less than 16 were suppressed during the calculation of incidence rates per standard US convention to protect patient confidentiality.

Unadjusted survival probabilities and cumulative incidence functions were calculated using flexible parametric survival models with 5 degrees of freedom for the baseline log cumulative hazard. The period of diagnosis was modeled as a time‐dependent variable to relax the proportional hazards assumption. All the study covariates were included in the multivariable models. Missing data were handled using multiple imputation with chained equations. *p*‐values < 0.05 were considered significant. Analyses were performed using STATA version 18.0.

## 3 Results

### 3.1 Patient Characteristics

There were 236 patients included in the study. There were 82 patients diagnosed between 2001–2010 and 154 patients diagnosed between 2011–2021. The median age at diagnosis for the entire study population was 51 years [interquartile range (IQR), 41–68]. There was a strong male predominance (*n* = 208, 88%), and most patients were White (*n* = 171, 72%). There were 140 patients (59%) with a documented history of chemotherapy for their lymphoma. Among these patients who received chemotherapy, the median time from diagnosis to receipt of chemotherapy was 16 days (IQR, 8–31). Table 1 shows the patient characteristics stratified by period of diagnosis. The characteristics were similar in terms of age at diagnosis, sex, race, and documented history of chemotherapy. The most frequent missing data was documentation of B‐symptoms at diagnosis for *n* = 71 (30%) of patients.

**TABLE 1:** Characteristics of patients diagnosed with primary effusion lymphoma in the United States, 2001–2021.

	2001–2010 *N* = 82 (%)	2011–2021 *N* = 154 (%)	*p*‐value
Median age at diagnosis [(IQR)], y	49 (41–62)	53 (40–74)	0.41
Sex			0.76
Female	< 16	19 (12%)	
Male	73 (89%)	135 (88%)	
Race			0.13
White	62 (76%)	109 (71%)	
Black	19 (23%)	31 (20%)	
Other	< 16	11 (7%)	
B Symptoms			< 0.001
No B symptoms	< 16	70 (45%)	
B Symptoms	30 (37%)	62 (40%)	
Chemotherapy			0.86
No/Unknown	34 (41%)	62 (40%)	
Yes	48 (59%)	92 (60%)	
Time from diagnosis to treatment, d	11 (6–24)	18 (10–33)	0.03

Abbreviation: IQR = interquartile range.

### 3.2 Age‐Adjusted Incidence Rates

Age‐adjusted incidence rates increased significantly between time periods. The age‐adjusted incidence rate was 1.0 cases (95% CI, 0.8–1.3) per 10,000,000 person‐years between 2001–2010. The age‐adjusted incidence rate increased to 1.6 cases (95% CI, 1.3–1.9) per 10,000,000 person‐years between 2011–2021. The corresponding incidence rate ratio was 1.52 (95% CI, 1.15–2.01; *p* = 0.004). Table 2 shows the estimated incidence rates by sex and time period. Men were 10‐times more likely to be diagnosed with the disease during the most recent time period.

**TABLE 2:** Age‐adjusted incidence of primary effusion lymphoma in the United States, 2001–2021.^a^

	2001–2010		2011–2021		(2001–2010) to (2011–2021)	
	*n*	IR (95% CI)	*n*	IR (95% CI)	IRR (95% CI)	*p*‐value
Total	82	1.0 (0.8–1.3)	154	1.6 (1.3–1.9)	1.52 (1.15–2.01)	0.003
Male	73	2.0 (1.6–2.6)	135	3.0 (2.5–3.5)	1.46 (1.09–1.98)	0.01
Female	< 16	^b^	19	0.3 (0.2–0.5)	^b^	^b^

Abbreviations: CI = confidence interval, IR = incidence rate, IRR = incidence rate ratio.

^a^ Incidence rates are age‐adjusted to the 2000 US standard population and expressed per 10,000,000 person‐years. Incidence rate ratios are based on unrounded rates.

^b^ These values could not be estimated due to < 16 cases.

### 3.3 Unadjusted Survival Estimates

**FIGURE 1:**
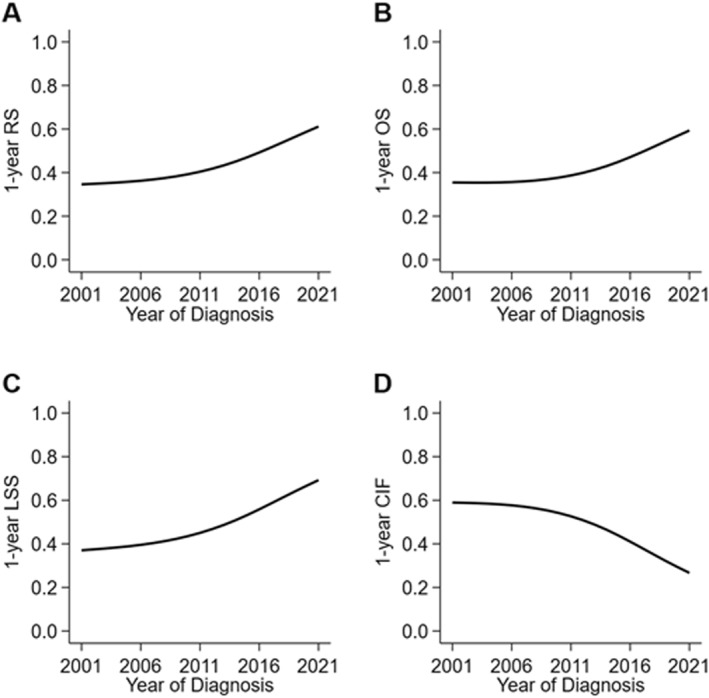
Survival trends for patients diagnosed with primary effusion lymphoma in the United States, 2001–2021. Trends in 1‐year outcomes are shown for (A) relative survival, (B) overall survival, (C) lymphoma‐specific survival, and (D) cumulative incidence of death.

Unadjusted 5‐year RS increased from 21% (95% CI, 13%–30%) between 2001–2010 to 37% (95% CI, 29%–45%) between 2011–2021. Five‐year OS increased from 18% (95% CI, 11%–25%) to 32% (95% CI, 25%–40%). Median OS improved from 4 months (95% CI, 2–6) to 12 months (95% CI, 5–20). Five‐year LSS increased from 21% (95% CI, 13%–30%) to 44% (95% CI, 35%–53%). On competing risks analysis, considering deaths from other causes, the 5‐year cumulative incidence of death from lymphoma decreased from 75% (95% CI, 66%–83%) to 49% (95% CI, 41%–57%). Figure 1 shows the unadjusted survival estimates over time demonstrating marked improvements in survival within the first year of diagnosis.

### 3.4 Multivariable Survival Models

On univariable analysis, the period of diagnosis was a significant predictor of outcomes with hazard ratios ranging from 0.49 to 0.63 (Table 3). On multivariable analysis after adjustment for age, sex, race, stage, documented presence of B‐symptoms at diagnosis, and documented receipt of chemotherapy, the period of diagnosis remained a significant predictor of outcomes. There was a 35% risk reduction in the overall mortality rate (OS, HR 0.65; 95% CI, 0.44–0.97; *p* = 0.03) and a 44% risk reduction in the lymphoma specific mortality rate (LSS, HR 0.56; 95% CI, 0.36–0.86; *p* = 0.01). For the lymphoma‐specific CIF, there was a 51% improvement in the sub‐distribution hazard rate (CIF, HR 0.49; 95% CI, 0.33–0.74; *p* = 0.001).

**TABLE 3:** Univariable and multivariable flexible parametric survival models.

Outcome	Unadjusted HR (95% CI)	*p*‐value	Adjusted HR (95% CI)^a^	*p*‐value
RS				
2001–2010	Reference		Reference	
2011–2021	0.63 (0.43–0.90)	0.01	0.67 (0.44–1.01)	0.06
OS				
2001–2010	Reference		Reference	
2011–2021	0.63 (0.45–0.90)	0.01	0.65 (0.44–0.97)	0.03
LSS				
2001–2010	Reference		Reference	
2011–2021	0.52 (0.35–0.77)	0.001	0.56 (0.36–0.86)	0.01
CIF				
2001–2010	Reference		Reference	
2011–2021	0.49 (0.35–0.68)	< 0.001	0.49 (0.33–0.74)	0.001

Abbreviations: CIF = cumulative incidence of death from lymphoma, LSS = lymphoma‐specific survival, OS = overall survival, RS = relative survival.

^a^ Models were adjusted for age, sex, race, stage, documented presence of B‐symptoms at diagnosis, and documented receipt of chemotherapy.

## 4 Discussion

The rarity of PEL makes clinical analyses challenging, with current literature consisting predominantly of case reports/series, institutional cohorts, and only small‐scale population‐based registry studies [2, 5, 6, 7, 8, 9, 10, 11, 12, 13, 14, 15]. Our analysis, utilizing the SEER‐17 database, a comprehensive population‐based cancer surveillance system, represents the largest population‐level evaluation of PEL survival outcomes to date. Herein, we report several important observations. First, there is a significant increase in age‐adjusted incidence rates from 1.0 to 1.6 cases per 10,000,000 person‐years in time period 2 (2011–2021) compared to time period 1 (2001–2010). Second, there is a significant improvement in survival outcomes in the most recent time period, with 5‐year overall survival nearly doubling and corresponding improvements in relative survival. Third, there is significant improvement in lymphoma‐specific survival on competing risk analysis, with notable reduction in the cumulative incidence of death from lymphoma.

Though our findings suggest progress in PEL outcomes, the increased incidence in time period‐2 compared to time period‐1 may reflect better disease recognition and diagnostic accuracy rather than true increased incidence, particularly given that our analysis begins when PEL was first introduced as a distinct entity in the WHO classification. Additionally, the marked male prevalence in disease distribution, with men being 10 times more likely to be diagnosed in the recent period, aligns with previously reported studies demonstrating consistent male predominance [2, 6, 9, 13, 14, 15, 16], and likely reflects the epidemiological overlap with HIV and other HHV‐8‐related malignancies.

Our population‐level findings align with the established literature. Earlier studies consistently reported poor outcomes, with median OS ranging between 4 and 6 months [3, 6, 8, 9, 17], comparable to our observed median OS of 4 months in time period‐1. More recent studies report improved survival, with median OS ranging from 7.6 to 10.2 months [10, 13, 18], closer to our observed population‐level median OS of 12 months in the recent period. Interestingly, a systematic review by Aguilar et al. including 299 cases through 2019 reported a pooled median survival of 6 months and found that survival improved over time in HIV‐positive cases from 1995–2019, similar to our reported improvement in overall survival in time period‐2 [2].

The improved survival outcomes likely reflect advances in the management of both lymphoma and HIV. Similar to other HIV‐related NHL, antiretroviral therapy (ART) is fundamental to the management of PEL, with retrospective studies showing remission when combined with chemotherapy regimens such as cyclophosphamide, doxorubicin, vincristine, prednisone/etoposide (CHOP/EPOCH), with rituximab added to CHOP for rare CD20 positive PEL. Among patients receiving chemotherapy, median survival ranges from 13 to 42.5 months [5, 13, 15, 19], with dose adjusted‐EPOCH achieving 3‐year cancer‐specific survival of 47% [19]. The highest reported median survival of 42.5 months was reported in a cohort that was mainly HIV‐positive and heavily treated with EPOCH regimens [15], supporting the effect of modern treatment approaches. Bortezomib‐based combinations also show improved outcomes when incorporated with concurrent optimal ART [11, 14]. Additionally, Brentuximab vedotin has shown single‐agent activity in relapsed/refractory PEL, with sustained complete remissions in multiply relapsed patients [20]. Novel approaches, including intracavitary cidofovir, radiotherapy, and autologous stem cell transplantation, have also been explored with varying success [21, 22, 23]. Beyond these advances, improved outcomes may also be attributed to modern approaches to HIV management, including increased recognition of HIV‐related complications leading to more timely diagnosis, and broader implementation of contemporary ART regimens which have substantially improved clinical outcomes and treatment adherence [24, 25].

Despite the observed improvement in survival, outcomes remain poor, particularly when compared to trends in other HIV‐associated lymphomas [9], with most evidence for treatment coming from small‐scale retrospective analyses or case reports. Additionally, treatment approaches have largely been adapted from HIV‐associated NHL regimens. Even less is known about PEL management in HIV‐negative patients. For immunocompromised patients, reduction of immunosuppressive therapies when possible has been suggested, however there is no evidence to support this.

Our study has several limitations. The SEER database does not capture detailed information about HIV status, CD4 count, HHV8 status, and specific treatment regimens are not available, limiting our ability to granularly assess the survival improvements observed. Additionally, the database may not capture extracavitary variants of PEL.

## 5 Conclusion

In conclusion, our findings show significant improvements in PEL outcomes over the past two decades, likely reflecting advances in both diagnosis and treatment of PEL. Despite this improvement, the OS continues to remain poor (median OS of 12 months) representing an unmet need. Our findings emphasize the need for prioritizing these patients to clinical trials with novel therapies.

## Funding

The authors have nothing to report.

## Consent

Informed consent was waived. The study was conducted in compliance with the Declaration of Helsinki. Since this was a retrospective study using publicly available data, the study was exempt from Institutional Board Review and informed consent was waived.

## Conflicts of Interest

The authors declare no conflicts of interest.

## Data Availability

The data that support the findings of this study are available from the corresponding author upon reasonable request.
